# Systematic Review of Gaming and Neuropsychological Assessment of Social Cognition

**DOI:** 10.1007/s11065-023-09599-y

**Published:** 2023-09-05

**Authors:** Elodie Hurel, Marie Grall-Bronnec, Orianne Bouillard, Marion Chirio-Espitalier, Malcolm Barrangou-Poueys-Darlas, Gaëlle Challet-Bouju

**Affiliations:** 1 CHU Nantes, UIC Psychiatrie et Santé Mentale, Nantes Université, Nantes, F-44000 France; 2 MethodS in Patient-centered outcomes and HEalth ResEarch, SPHERE, Nantes Université, Univ Tours, CHU Nantes, INSERM, Nantes, F-44000 France

**Keywords:** Video games, Gaming disorder, Social cognition, Neuropsychology, Problematic gaming

## Abstract

Playing video games is associated with cognitive changes and possibly psychosocial difficulties. Problematic gaming occurs upon the loss of control over videogame playing; gaming disorder is considered a behavioral addiction in the 11th version of the International Classification of Diseases. Models used to understand behavioral addictions include cognition as an essential factor in the development, maintenance, and relapse of addiction. Nevertheless, some aspects of cognition, such as social cognition, remain underexplored, despite evidence of alterations in cognitive and social function among patients with problematic gaming. This review aimed to describe the current understanding of social cognition in individuals exposed to videogames. We included all studies assessing social cognition in participants of any age with a wide range of exposure to video games (from simple use of video games (such as at least two exposures) to problematic gaming, defined according to the included study). This wide range of exposure allowed us to explore the whole process from repeated exposure to addiction. We included only studies that used neuropsychological tasks to assess social cognition. Patient-reported outcomes that could be biased by subjective self-report data were not included. The search was conducted from inception to January 2022 in three databases (PubMed, PsycINFO, and Web of Science). The systematic search identified 39 studies that assessed facial emotion processing, empathy, theory of mind, social decision-making, aggressive behavior, and moral competence. In general, results have been mixed, and a number of questions remain unanswered. Nevertheless, several studies showed cerebral changes when processing facial emotion that were linked with problematic gaming, while no link was obtained between nonproblematic gaming and empathy alterations. The influences of cooperation patterns, theory of mind, moral competence, and gaming frequency were highlighted. Finally, there was substantial heterogeneity in the population assessed and the methods used.

## Introduction

### Video Gaming

Video gaming is a modern leisure activity that refers to the use of video games (VGs), such as massively multiplayer online role-playing games (MMORPGs), multiplayer online battle arenas (MOBAs), first-person shooters (FPSs), or even sandbox games (games without planned scenarios) (Leouzon et al., 2019). Gamers can play for entertainment but also to deal with anxiety, depression, or other psychological difficulties. Nevertheless, the causality of the relationship between motives/psychological difficulties and gaming is poorly understood (Sunil et al., 2021). Playing VGs has been found to increase the activity of the sympathetic nervous system (Krarup & Krarup, 2020). Gaming also leads to brain changes (Brilliant et al. 2019) and enhances aspects of cognition, such as top-down attention processes and spatial cognition (Bediou et al., 2018). Regarding social function, time spent playing VGs is negatively associated with the quality of social relationships (Lo et al., 2005). In contrast, psychological well-being and social functioning were positively linked with VG use in a population of heavy gamers (van den Eijnden et al., 2018). Gamers also have high online social capital (i.e., networking and its resulting benefits (Williams, 2006; Collins & Freeman, 2013).

### Problematic Gaming and Gaming Disorder

The 11th version of the International Classification of Diseases (ICD-11) (World Health Organization, 2018) defines gaming disorder (GD) as “impaired control over gaming, increased priority given to gaming over other activities to the extent that gaming takes precedence over other interests and daily activities, and continuation or escalation of gaming despite the occurrence of negative consequences.” Another clinical definition was provided for Internet gaming disorder (IGD) in the Diagnostic and Statistical Manual of Mental Disorders, 5th edition (DSM-5), within the section outlining disorders requiring further investigation (American Psychiatry Association, 2013). Both IGD (in the DSM-5) and GD (in the ICD-11) refer to similar disorders characterized by a loss of control over video gaming leading to negative consequences but have slight differences in operationalization for diagnosis.

In the present review, we use the umbrella term “problematic gaming” (PG) to encompass the variety of clinical definitions used in the studies reviewed. PG includes the clinical definitions provided in the international classifications (IGD in the DSM-5 and GD in the ICD-11) as well as other definitions used before the development of the ICD-11 or DSM-5 or within other conceptual frameworks used to characterize excessive and harmful use of VGs (Schettler et al., 2022). In the present review, the term PG is contrasted with nonproblematic gaming (NPG) which is defined as leisure activity without a loss of control or harmful consequences.

The prevalence of PG is estimated to be between 0.7 and 27.5% in the general or gamer population, depending on the clinical definition used, with more men affected (Mihara & Higuchi, 2017).

Models of the underlying processes of addictions are multifactorial, and these processes include cognitive functioning, among other factors (Brand et al., 2016, 2019; Noel et al., 2013). Indeed, alterations in inhibitory control (Argyriou et al., 2017) and in frontostriatal and frontocingulate networks (Yao et al., 2017) have been highlighted in people with PG. Moreover, improvements in PG symptoms have been linked to improvements in cognitive functioning (Lim et al., 2016). In contrast, NPG has been linked with improvements in cerebral structures and connectivity and optimization of neuronal recruitment, especially for attention and visuospatial skills (Palaus et al., 2017).

Regarding social functioning, PG was found to negatively impact social relationships (Ryu et al., 2018). More specifically, individuals with PG have high online social capital, as do gamers without PG, but low offline social capital (Collins & Freeman, 2013). Additionally, psychological well-being and social functioning were negatively linked with the presence of PG (van den Eijnden et al., 2018) and were even a predictor of it (Lemmens et al., 2011). The presence of PG has also been linked to familial (Hyun et al., 2015), emotional, and behavioral problems (Frölich et al., 2016). Taken together, these findings suggest an alteration in social functioning in people with PG.

### Definition of Social Cognition

The literature suggests the presence of altered social functioning among individuals with PG. Nevertheless, gamers’ social functioning is less understood for those without PG. On the borderline between cognition and social functioning, there is social cognition (SC). This concept includes all cognitive processes underlying social interactions, from the ability to detect and identify social cues, to interpret social cues, to the generation of socially appropriate responses (Adolphs, 2001; Frith, 2008; Saxe, 2006). SC also allows people to construct social norms that will serve as standards for future social interactions (Beer & Ochsner, 2006; Bertoux, 2016; Greifeneder et al., 2017). Indeed, given these social abilities, the “social brain” theory postulates that the human brain is larger than that of other species because of the complexity of our social world and, specifically, our ability to bond with others (Adolphs, 2009). The crucial role of SC in daily functioning has been demonstrated in several psychiatric clinical populations, such as patients who suffer from schizophrenia (Brunet et al., 2003; Couture et al., 2006).

### Components of Social Cognition

SC covers a wide spectrum of cognitive functions. SC tasks can range from the simplest tasks (e.g., recognizing an emotion on a face) to the most complex (e.g., understanding irony or social decision-making). Several models have been constructed to determine the number of SC components (Fiske & Taylor, 2016; Green et al., 2015; Happé & Conway, 2016). For example, it is debated whether the self should be included in this concept (Happé & Conway, 2016). Similarly, the inclusion of alexithymia (which reflects the inability to identify and describe emotions experienced by oneself or others (Etchepare & Prouteau 2017; Taylor et al., 1985) within the spectrum of SC is controversial. Several authors have also proposed differentiating SC components based on more general characteristics. For example, the level of processing (e.g., high, low or dual processing (Adolphs, 2009, 2010; Etchepare & Prouteau, 2017; Frith & Frith, 2008) or the nature of the stimuli involved (affective versus cognitive stimuli) may be used (Adolphs, 2010; Etchepare & Prouteau, 2017; McDonald, 2013; Shamay-Tsoory & Aharon-Peretz, 2007).

In the following paragraphs, only SC components identified in the literature and described with systematic research are presented for the sake of clarity.

#### Social Information/Emotion Processing, Emotion Attribution, and Affective Cognition

The study of social or emotional processing can occur at two levels. The conscious level is assessed by asking the subject to identify an emotion or rate its intensity, for example. The unconscious level can be measured when the participant attends to another task while faces are presented. Facial emotion processing seems to be supported by two networks. The ventral network deals with static stimuli and associated stimuli with information such as the identity of the face. The dorsal network deals with dynamic stimuli (Duchaine & Yovel, 2015). The ability to process emotions and make assumptions about them is also called affective cognition (Ong et al., 2015).

#### Empathy

Empathy is the ability to feel what another person is feeling and understand him or her (Lockwood, 2016). Several models of empathy have been proposed. For example, one model postulates that empathy has two components. The first component involves the ability to understand motives and thoughts from another person (i.e., *cognitive empathy*). The second component involves the ability to experience the feelings of another person (i.e., *emotional empathy*) (S. Shamay-Tsoory et al., 2009). Another model separates empathy into two different components. The first component, *personal distress*, is self-oriented and reflects the tendency to try to alleviate one’s pain. The second component, *compassion* (or *empathic concern*), involves the ability to feel sympathy for others (Singer & Klimecki, 2014). The function of empathy, also called vicarious experience, seems to be supported by the anterior cingulate cortex and the anterior insula (Lockwood, 2016; Singer & Klimecki, 2014).

#### Theory of Mind, Attribution Style/Bias, and Intention Attribution

Another component that is sometimes considered similar to affective empathy (Decety & Lamm, 2006; Lockwood, 2016) is *theory of mind* (ToM). ToM is also called mentalizing, mind reading, or cognitive perspective (Singer & Klimecki, 2014). It reflects the ability to infer mental states (*cognitive ToM*) or emotional states (*affective ToM*) of another person (Shamay-Tsoory & Aharon-Peretz, 2007). It allows individuals to understand and predict the behavior of others (Premack & Woodruff, 1978). ToM seems to be supported by a network including at least the medial prefrontal cortex (mPFC) and bilateral temporoparietal junctions (Happé & Conway, 2016; Schurz et al., 2014). Attribution style is defined as “how people deduce causal relationships and characteristics of other persons in the environment” (Fiske & Taylor, 2016). Attribution style is linked to the concept of ToM and allows the observation of hostile attribution bias, as found in individuals with schizophrenia (Buck et al., 2020).

#### Social Decision-Making

On another level of reasoning, *social decision-making* is the ability to make a decision that will affect oneself and another person, with the outcome depending on the behavior of oneself and another person. This function seems to be supported by the prefrontal cortex but also by the amygdala and insula, which handle affective biases that impact decision-making (Rilling & Sanfey, 2011).

#### Aggressive Behavior

Aggressive behavior involves the administration of an unpleasant stimulus to another person (Taylor, 1967). Cognitive activity associated with this behavior seems to be linked with the dorsal part of the mPFC. The affective component (i.e., compassion during punishment) seems to be supported by the ventral part of the mPFC (Lotze et al., 2007).

#### Moral Decision-Making

Another component of SC is moral decision-making. This ability has 3 aspects: a particular predilection toward a moral orientation (affective component), the ability to make decisions regarding moral dilemmas based on those moral aspects (cognitive component, also called *moral competence*), and the fact that decision-making is not dependent on the issues in the particular situation (Jung et al., 2016; Lind, 2008). This ability is underpinned by emotional and cognitive processes that come into play in decision-making. For example, a higher level of moral competence has been linked to stronger functional connectivity between the amygdala and the ventral mPFC and weaker connectivity between the amygdala and frontoparietal control network (Jung et al., 2016).

#### Social Knowledge

Social knowledge refers to all the information stored in memory that can be used to infer mental states. This information can be general, such as norms about how to behave in certain situations or places. Information may also be specific to the person with whom the interaction takes place such as history with that person (Achim et al., 2013; Langdon et al., 2014).

### Social Cognition and Gaming

The aim of this review is to describe the current understanding of the relationship between SC and gaming. Indeed, it seems that NPG, and, to a greater extent, PG, can impact both cognitive and social functioning (e.g., building relationships); thus, they may also impact SC (e.g., identification of facial emotions). Furthermore poor cognitive functioning, specifically poor SC, may lead to increased time spent gaming and even PG. As SC involves both cognition and social functioning, it is logical to explore the literature on SC in gamers to elucidate the sociocognitive profiles of gamers with or without PG. This review will also allow us to identify the SC competencies involved.

## Methods

### Search Strategy

The search strategy was developed in accordance with the Preferred Reporting Items for Systematic Reviews and Meta-analyses (PRISMA) recommendations (Moher et al., 2009). The search was conducted from database inception through January 2022. No registration was made, and no protocol was prepared. We initially planned to register the protocol of this literature review ahead of time with the PROSPERO registry. However, the registration was not accepted because the data explored were not considered related to a health condition (Moher et al., 2014). The search was performed in three databases (PubMed, PsycINFO, and Web of Science). Key words regarding SC and VGs were crossed (see Table 1), resulting in the following search: (“video game” OR “video games” OR “videogame” OR “videogames” OR “game” OR “games” OR “gaming” OR “gamer”) AND (“mentalization” OR “mentalizing” OR “mentalising” OR “mind reading” OR “social cogniti*” OR “social interaction” OR “social function*” OR “social brain” OR “social decision” OR “social perception” OR “affective cognition” OR “social knowledge” OR “social information processing” OR “emotion attribution” OR “attributional style” OR “theory of mind” OR “emotion processing” OR “attribution bias” OR “intention attribution” OR “empathy”). The SC-related keywords were determined based on the current literature on SC, regardless of the theoretical model, to scan a wide variety of SC components. The search focused on articles published in French or English.

**Table 1:** Keywords used in the database search

Gaming keywords	Social cognition keywords
Video game(s)/videogame(s)	Social function(ing)
Gaming	Social cognition/social cognitive
Game(s)	Social interaction
	Social brain
	Social perception
	Social information processing
	Affective cognition
	Emotion attribution
	Emotion processing
	Empathy
	Mentalization/mentalising/mentalizing
	Mind reading
	Attributional style
	Theory of mind
	Attribution bias
	Intention attribution
	Social decision
	Social knowledge

### Inclusion and Exclusion Criteria

This systematic review focused on both NPG and PG. As SC in VGs is a recent research area, we chose not to restrict our review to any specific pattern of gaming exposure (e.g., early exposure during childhood, training people with no VG experience, or controlled studies of people with and without VG experience) or any level of exposure (e.g., frequency of VG playing). However, we did not include studies that did not assess the frequency of VG exposure or did not expose participants to VGs more than once. Indeed, a single exposure to VGs is insufficient to induce changes in cognitive functioning, as the repetition of an action (harmful or therapeutic) is necessary. This strategy allowed us to explore the whole process from repeated exposure to addiction and to identify the areas in which the literature was the most developed and, conversely, the areas in which additional research is needed.

Moreover, only assessments of SC based on neuropsychological performance (i.e., performance-based tasks objectively linking behavior and the brain (Casaletto & Heaton, 2017) were included in this review. Studies using self-reported outcomes, which are subjective assessments made by the participant (Rothman et al., 2007), were not included. Indeed, self-reported data are subjective, and they do not allow an evaluation of the quality of cognition.

Articles were included if they (i) evaluated participants’ frequency of gaming OR the effect of playing VGs more than once OR patients with PG AND explored its impact and (ii) included a neuropsychological task to assess SC.

### Study Selection

After the exclusion of duplicates, each title was screened by the first author and excluded if it was clearly not within the scope of the review (i.e., articles not considering VG as a leisure activity or not assessing cognition). This selection process resulted in a large sample of abstracts that were screened twice. The first reading by the first author excluded abstracts that were clearly off-topic. The second selection of abstracts was performed using Abstrackr software (Wallace et al., 2012) by the first and fifth authors. This software can be used to save time by screening titles and abstracts based on machine learning that classifies titles as relevant or not (Rathbone et al., 2015). We used it only as a collaborative tool that was accessible online.

Because of the lack of information in abstracts, a large number of articles were retained during this abstract screening step. The methods of these articles were screened by the first and fifth authors to ensure that we did not miss any study that could have included neuropsychological SC tasks. Therefore, we read the methods of all articles dealing with cognition in gaming, even without apparent SC assessments and/or apparent exclusive use of self-rated questionnaires. Finally, the full texts of studies were read, and studies were included in accordance with the inclusion criteria. To complete the database search, the first author performed a manual search and screening of the bibliographic references of the studies included and from a recently published meta-analysis regarding gaming and social outcomes (Greitemeyer & Mügge, 2014).

### Data Extraction

Each full text that was considered eligible was read by the first author. Information about the study population, measures of VG use or PG, cognitive assessment of SC, and main results exploring the effect of VGS are presented in Table 3. Table 3 also provides information on the power of the studies reviewed, by categorizing studies according to their methodology (comparative or correlational studies). A summary is provided at the beginning of the “Results” section to describe demographic information, SC outcomes, and VG information. In the interpretation of the results, we were careful to differentiate the results from samples of gamers with and without PG. Indeed, this may provide information on the links of SC with NPG versus PG.

## Results

**Fig. 1:**
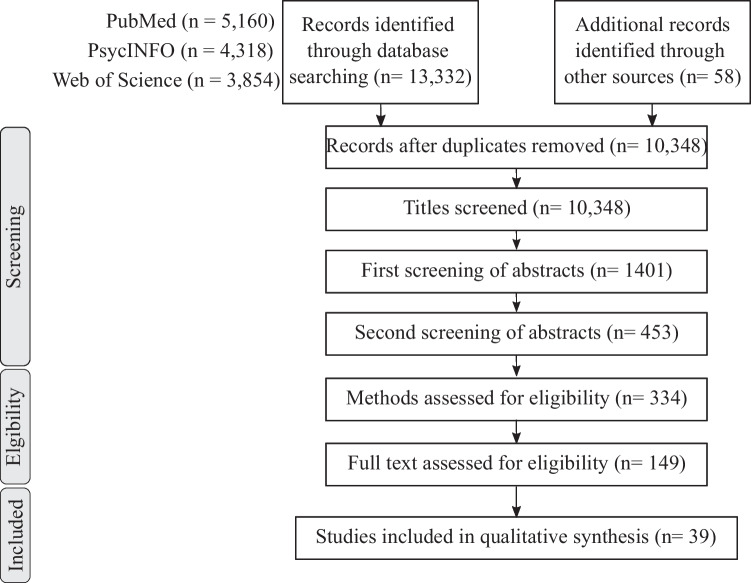
Flowchart of the systematic review

Research included in the previously published meta-analysis (Greitemeyer & Mügge, 2014) led to the screening of 52 additional records. From these 52 studies, 34 were screened based on their abstract, 15 were then screened based on their full text, and 3 were finally included. All numbers and reasons for exclusion at each step are included in Table 2 and Fig. 1. Moreover, all references from articles included in the systematic review and previously published relevant meta-analyses (*n* = 23) were also screened, and four articles were included from these lists. In total, the systematic search resulted in the selection of 39 studies.

**Table 2:** Reasons for exclusion at each step of screening

**Reasons for exclusion based on titles (*n* = 8947)**	
Not an original research article	282
Videogame (VG) was used as a tool or a serious game	2664
Not about gaming	5618
Not about cognition	384
**Reasons for exclusion based on abstracts — first reading (*n* = 948)**	
Not an experimental research article	88
Not published in English or French	1
Not about gaming	56
VG is used as a tool or a serious game	55
Not focused on cognition	572
Single exposure and/or assessment of frequency of gaming	19
Not focused on social cognition	157
**Reasons for exclusion based on abstracts — second reading (*n* = 119)**	
Not an experimental research article	8
Not published in English or French	2
VG is used as a tool or a serious game	16
Not about gaming	5
Not focused on social cognition	22
Not focused on cognition	66
**Reasons for exclusion based on methods (*n* = 185)**	
Not an experimental research article	8
Not published in English or French	3
VG is used as a tool or a serious game	2
Not about gaming	3
Not focused on cognition	93
Not focused on social cognition	76
**Reasons for exclusion based on full texts (*n* = 110)**	
VG is used as a tool or a serious game	2
Single exposure with no assessment of frequency of gaming	32
Not an experimental research article	13
No social cognition (SC) neuropsychological task	38
Frequency of gaming was assessed but not used to study its impact on SC	18
Only short-term exposure to VG was assessed	7

### Results Summary

In these 39 studies, the average sample size was approximately 230 (min = 28, max = 3,034, median = 99, average sample size of comparative studies = 73, average sample size of correlational studies = 342).

The mean age of participants included in these studies was 20.13 years (min = 5.06, max = 32.04, 3 studies with missing data, mean age of comparative studies = 22.12, mean age of correlational studies = 18.78). Participants were mostly male (59.64%) (2 studies with missing data).

Within the 39 studies, 23 different SC outcome measures were used to assess 6 components of SC (facial emotion processing, empathy, ToM, social decision-making, aggressive behavior, and moral competence). Of the 39 studies, 27 found significant results regarding SC outcome measures (11 on facial emotion processing, 1 on empathy, 6 on ToM, 2 on social decision-making, 5 on aggressive behavior, and 2 on moral competency).

Regarding VG information, only 13 experiments of 43 provided information regarding the type of VG played by gamers (8 assessing facial emotion processing, 2 assessing empathy, 2 assessing ToM, and 1 assessing social decision-making). Moreover, a large number of experiments (32 of 43) focused on exposure to violent VGs. Among them, 19 found significant results (8/11 on facial emotion processing, 1/5 on empathy, 4/7 on ToM, 2/2 on social decision-making, and 4/7 on aggressive behavior). Additionally, 11 studies included frequent video gamers, 4 included addicted video gamers, and 27 included individuals from the general population (23 assessed the frequency of gaming, 3 exposed to VGs, and 1 assessed the severity of addiction). Among the 15 studies assessing at least regular gamers, 12/15 (80%) reported significant results in the domains of facial and social emotion processing (*n* = 7), social decision-making (*n* = 2), aggressive behavior (*n* = 2), and moral competence (*n* = 1). Among the studies conducted in the general population, 16/28 (57%) found significant results (5 assessing facial emotion processing, 1 assessing ToM, 6 assessing empathy, 3 assessing aggressive behavior, and 1 assessing moral competency). Finally, 19 studies included a control group. Among them, 13 (68%) found significant results, while 15/24 (63%) studies without a control group showed significant results.

Regarding the assessment of addicted gamers, three studies used the Internet Addiction Test (IAT (Young, 2016)), and one of them used the DSM-5 criteria to classify participants. All three reported significant results.

### Detailed Results on Each SC Domain

To facilitate understanding, the articles are presented according to the various components of SC assessed and ranked from the most recent to the oldest in Table 3.

**Table 3:** Description of articles found in the systematic search

**First author, year of publication**	**Population assessed**	**Group characteristics**	**Gaming information**	**SC measure**	**Main results**
**Facial emotion processing**					
	*Group comparisons*				
Pichon et al. (2021)	Study 1: prticipants that answered local ads Study 2: university students	Study 1: 47 VG players (AVGP): mean age = 22.3, SD = 4.3; 50 non-VG players (NVGP): mean age = 24.2, SD = 5.1 Study 2: 27 AVGPs (mean age = 22.81, SD = 2.69); 27 NVGP (mean age = 22.96, SD = 3.66)	AVGPs: played 5 h per week of FPSs and 1–5 h per week of another VG. NVGPs: played at most 5 h per week across all games possible and 1 h at most of each possible VG	Study 1: face stimuli: morphing expression over a sequence of 15 steps, from sad to angry and from pain to happy as well as from neutral to pain, happy, fear, or sadness in session 2. Participants had to identify the emotion displayed. Study 2: facial animation of 3 facial muscles randomly chosen among 42 possible action muscles. After each animation, participants had to categorize the emotion displayed among 7 possible choices (happy, sad, angry, fearful, surprised, disgusted or other), and had to rate the intensity of emotion	Study 1: no difference between groups in emotional identification in both sessions. Study 2: no group difference in discrimination
Kang et al. (2020)	Professionals gamers (e-sport players group; EPG) and professional baseball players (pro-baseball group; PBG)	EPG: *n* = 55, mean age = 21.3, SD = 1.4; PBG: *n* = 57, mean age = 21.3, SD = 1.4. Control group (CG) from the general population matched on sex, *n* = 60, mean age = 21.3, SD = 1.5	EPG: included an elite group (*n* = 12) that participated in 80% of games each season; PBG: included an elite group that attended 144 major league games out of 144 or that had 446 plate appearances	Emotion perception test: 108 trials with 2–8 faces displayed on the screen. Participants had to indicate if all faces presented displayed the same emotion or not	The PBG group displayed faster reaction times than the EPG and the CG. The elite group in the EPG displayed faster reaction times on the emotion perception test than the rest of the EPG group
Jiang et al. (2020)	Patients from a mental health facility and citizens of Wuxi	Internet gaming disorder (IGD) group: *n* = 30, mean age = 22, SD = 5, 53.3% male. Methamphetamine (MD) group: *n* = 30, mean age = 22, SD = 6, 43.3% male. Normal control (NC) group: *n* = 30, mean age = 22, SD = 6, 50% males	The IGD group met the IGD criteria of the DSM-5; the MD group met the criteria of MD dependence according to the DSM-5, had a cumulative MD use of over 50 mg, and spent less than 2 h per day on the Internet. The NC group excluded smokers, alcohol use disorder or substance dependence; participants spent less than 2 h per day on the Internet	The interpersonal perception task (IPT-15) was used to measure social perception. It consists of 20 min of video of 15 scenes including one to four roles. Five social roles were possible (kinship, intimacy, deception, competition, and status). Participants had to identify the correct role displayed in the scene	The IGD and MD groups had similar IPT-15 scores. Both groups scored lower than the NC group
He et al. (2019)	Undergraduate students from Wuhan University	PG group: *n* = 15, mean age = 20.97, SD = 1.65. Control group: *n* = 15, mean age = 21.02, SD = 1.70	PG group: IAT score ≥ 80, Internet gaming addiction scale (IGAS) score ≥ 7, mainly played “Strike of Kings,” and PG diagnosis according to a clinical interview led by two psychiatrists. Control group: IAT score ≤ 40, IGAS score ≤ 3, preferred to play “Strike of Kings”	Oddball paradigm: participants indicated the change in size of the fixation cross while pictures of faces (humans or characters from the VG “Strike of Kings”) appeared. Participants were to ignore the faces. Two blocs were included, one with humans as standard stimuli and characters as deviant stimuli (with a ratio of 8:2) and vice versa. EEG data were continually recorded	Analyses showed that, for character faces, the PG displayed enhanced P100, P200, and MMN components, and an absence of left dominance of the N170 component. The PG group also displayed an advantage when processing characters
Kühn et al. (2019)	Participants from the general population	Violent video game (VVG) group:* n* = 25, mean age = 26.6 years, SD = 6.0, 14 females. Active control group: *n* = 24, mean age = 25.8 years, SD = 6.8, 12 females. Control group: *n* = 28, mean age = 30.9 years, SD = 8.4, 12 females	The VVG group played Grand Theft Auto 5 for 8 weeks, 30 min a day, while the active control group played Sims 3 for weeks, 30 min a day. The control group was not given a game to play	Reading the mind in the eyes task (RMET; attribution of complex emotions based on photographs of eyes) along with several other non-SC tasks	No effects of the VVG were was shown
Stockdale et al. (2017)	University students	Frequent players of violent, graphic VGs: *n* = 30. Infrequent players of violent, graphic VGs: *n* = 31. Mean age = 20.68, SD = .21	Frequent players played at least 30 h of VGs per week, with 2 violent VGs out of 3 VGs played. Infrequent players played no more than 5 h a week, with 2 nonviolent VGs out of 3 VGs	The stop signal task (SST) involved two emotions displayed by faces: happy or afraid. Participants were asked to press the button to indicate if the face presented was male or female and to refrain from pressing the button if a striped box appeared around the stimulus. The difficulty increases when participants produce correct answers. EEG data were continually recorded	SST results: no significant difference was found between groups Regarding the P100/N200 and P300 components, smaller amplitudes were observed in frequent players than infrequent players, specifically for happy faces. For the N170 component, infrequent players had shorter latencies to identify fearful facial expressions than for happy facial expressions Frequent players had shorter latencies for happy expressions than for fearful expression in go trials. In stop trials, frequent players had lower mean amplitudes than infrequent players
Peng et al. (2017)	Local university students of in Shenzhen, China	PG group: *n* = 16, mean age = 20.75, SD = 0.36. Control group (CG): *n* = 16, mean age = 20.24, SD = 0.4	PG group: participants with an IAT score ≥ 40 who spent at least 4 h a day and 30 h a week on Internet games and had scores of less than 40 points on the Zhung Self-Rating Depression Scale and the Zhung Self-Rating Anxiety Scale	Faces (neutral or happy/sad depending on the block) were displayed on the screen with a backward mask paradigm permitting the observation of unconscious processing. Participants had to indicate if the stimuli were emotional or neutral. EEG data were recorded	The CG was faster than the PG group on the sad block. Reduced amplitudes of the N170 component (reflecting facial processing) were observed for the PG group when processing neutral faces compared to happy ones, while the CG did not show any difference in this situation. No other difference between groups was found
Diaz et al. (2015)	Undergraduate students from the University of Calgary	*N* = 152, 73 males, mean age = 20.45, SD = 3.12	Prescreening was used to select participants playing 0 h of VVGs per day or at least 2 h per day of VVGs (realistic or fantasy, according to the Entertainment Software Rating Board)	A facial emotion identification task was used in which participants had to evaluate the emotion displayed by the face or the gender of the face (in the control condition). The intensity of emotion was low, medium or high	No overall effect of group was found. VVG players were more accurate and faster in identifying fearful faces than nongamers. Nongamers were more accurate in identifying disgusted faces than VVG players
Bailey and West (2013)	Students from Iowa State University	Nonexposure group: *n* = 9 (mean age = 24.22, SD = 8.43). Action group: *n* = 10 (mean age = 20.40, SD = 2.01). Nonaction group = 9 (mean age = 21.77, SD = 4.02)	Subjects reported playing 0 h of gaming per week and weekend at baseline. The nonaction group played Tetris for 1 h each day for 10 days, while the action group played an action game for the same amount of time. The nonexposure group did not play VGs. The three groups were assessed twice, separated by 10 days	The emotion search task was used; this task presents matrices with 9 faces to participants, who are instructed to decide whether an emotional face (angry or happy) is among the neutral faces. This task was performed with EEG recording	Attenuation of reaction times was found when comparing the nonexposure group to the nonaction group, with a diminution of difference in reaction time according to target presence for the nonaction group. Effects on cerebral processing were different between gaming groups. For example, there was a decrease in attention to happy faces in the action group (shorter latencies of the P3 in the action group between pretest and posttest), while the nonaction group showed differences in the processing of targets (difference in the P3 electrode on the right hemisphere, with a greater amplitude between pretest and posttest). An analysis of latent variables was also conducted (see the original article for details)
	*Correlational studies*				
Miedzobrodzka et al. (2021a)	High school students (study 1) and university students (study 2)	Study 1: *N* = 69, mean age = 17.06, SD = 0.69. Study 2: *N* = 151, mean age = 26.68, SD = 6.93)	Both studies: VVG exposure was computed according to rating of the frequency and violent content of VGs played	Both studies: the facial expressions matching test was used, which measures the ability to recognize negative facial expressions (disgust, fear, anger, sadness). Four faces were displayed. Participants were instructed to match the face in the center with the emotion of one of the three faces shown on the bottom	There was a negative relationship between VVG exposure and accuracy in the recognition of negative facial expressions in adolescents and adults
Aydin et al. (2020)	8th grade secondary school students	477 students	Internet gaming disorder test (IGDT): self-report of 6 subscales of IGD symptoms	RMET, child version: 28 photographs with only the region of the eyes were displayed. Participants had to choose the emotion displayed among four options	Poor negative emotion recognition was linked to PG symptoms and may be a vulnerability factor for PG
Bonny et al. (2020)	Players of MOBA (Dota 2) attending a tournament	*N* = 335 (31 females, 311 males), mean age = 23.418 years, SD = 4.075)	Participants provided a Steam account for statistics data. Experience was assessed by the number of matches completed. Player performance was assessed using the match making rating (MMR, calculated by the algorithm of the platform and not available) and the win–loss ratio (WLR). Several measures of gaming experience were recorded (T0 = at the tournament, T2 = at 3 months and T3 = at 4 months)	The RMET was used a measure of ToM. Only pictures of eyes were shown; participants had to decide which emotion was shown among four options. In the economic cooperation task, participants had to choose whether to cooperate with strangers for a fictional company. If both cooperated, the profit was higher than if both defected. If only one cooperates, their company had an economic disadvantage compared to the company of the participant that defected A task of elicited attention using a line drawn eye gaze cue was used to assessed attentional allocation to visual cues	RMET accuracy was a significant predictor of the MMR. Better RMET scores had a greater impact on the performance improvement in party (i.e., playing with familiar people) games than solo games There was no association between VG exposure and visual attention allocation Results of the economic task were not included in the analysis because of poor reliability
Miguel et al. (2017)	Participants recruited via online systems	*N* = 164, mean age = 18.93, SD = 6.36, min = 14, max = 59, 123 females	A game preference questionnaire was created to assess the frequency, type of game, and who players played with, etc	The computerized test of primary emotion perception (PEP) included 38 soundless videos in which actors displayed emotions that the participant had to identify	A positive correlation between the time spent playing VGs (in years) and the general perception of emotion were found. Moreover, a negative correlation between playing hours and attribution of joy was found; in other words, greater VG exposure led to worse identification of joy. A correlation between the frequency of meeting people to play and general emotion perception was highlighted, in which the more you met, the better the emotion identification
**Empathy**					
	*Group comparisons*				
Kühn et al. (2018)	General community, recruited using flyers and Internet advertising	*N* = 90, mean age = 28, SD = 7.3, range = 18–45, 48 females. VVG group: *n* = 26, mean age = 27, SD = 6.1, 14 females. Active game control group: *n* = 24, mean age = 26.1, SD = 6.9, 13 females. Passive control group: *n* = 30, mean age = 30.8, SD = 8.3, 13 females	No VG use in the past 6 months. Training consisted of playing VGs for eight weeks, approximately 30 min a day. The VVG group played Grand Theft Auto, the active control group played the Sims, and the passive control group did not play any VGs. Assessments took place before the gaming training (baseline), right after the 2 months of training (T1) and then 2 months later (T2)	Twenty-eight photographs of hands with tools were shown during fMRI scans; half of the photos contained painful contexts	No desensitization for pain (diminution of reaction to presentation of violent stimuli) was found
Szycik et al. (2017)		Only males. VVG group: *n* = 15, mean age = 22.8, SD = 4.3 years. Control group: *n* = 15, mean age = 22.1, SD = 3.0	The VVG group included participants who played FPSs for at least 4 years for 2 h daily. The control group reported no experience of playing VGs	Drawings with black lines (varying in emotional charge [yes/no] and social context [yes/no]) were presented for 4 s during fMRI scans. Participants were asked to look at them carefully and imagine how they would feel in that situation	No differences between groups were found
Gao et al. (2017)	Southwest University, China	20 students from the 25th percentile (low-exposure) and 20 students from the 75th percentile (high-exposure) of VG exposure. Age range: 18–27 years, mean age = 21.17, SD = 0.065. VG group: *n* = 18. Nongaming group, *n* = 17	Two hundred undergraduates completed the Anderson and Dill video game questionnaire in which they listed the VGs played and then rated the amount of violence in each VG and the frequency played. A score of VG exposure was then calculated	In the fMRI, painful or nonpainful pictures of hands were displayed. Participants were asked to concentrate and imagine the experience of the persons whose body parts were shown in the picture	No difference between groups was found
	*Correlational studies*				
Ferguson and Colwell (2020)	Mail and social media diffusion	125 participants (57 men), mean age = 26, SD = 11.2	Participants listed their three favorite VGs and rate the frequency of play of each. Each VG was rated in term of violence and sexualized content	A vignette of rape was presented to participants, and a scale of empathy toward the victim was used to assess feelings (Gabbiadini, 2016)	No effect of VVG or sexualized VG exposure on empathy toward the rape victim was found
Miedzobrodzka et al. (2021b)	University students	*N* = 58 (100% male), mean age = 22.41, SD = 3.42, right handed	VVG exposure was measured using the name of their three favorite games, the time spent on these games, and official violent content ratings	Pain judgment task: in this task, participants viewed pictures of hands in painful or nonpainful contexts, while EEG data were recorded. Participants had to categorize each picture as painful or not. Intensity of pain was rated within a second block	The P3 component (particularly sensitive to aversive stimuli) and the P625 are reflective of top-down responses to painful pictures. Regarding the P3: no difference was found between painful and nonpainful pictures in the high-exposure group, in contrast to the low-exposure group, suggesting desensitization. Regarding the P625: a difference between painful and nonpainful pictures (with higher amplitudes for painful pictures) was found in both groups
**Theory of mind — attribution bias**					
	*Correlational studies*				
Ferguson and Wang (2019)	Children	*N* = 3034. Mean age at T1 = 11.21, SD = 2.06, mean age at T3 = 13.12, SD = 2.13	VVG exposure was measured using the name of the three favorite games, the time spent on these games, and official violent content ratings	Hostile aggression bias was measured with six ambiguous scenes. Children had to rate the level of aggressive intent in each scene	No link between hostile bias and exposure to VVG was found
MacGowan and Schmidt (2021)	Children	73 typically developing (TD) children (39 female). Two assessments: mean age at T1 = 54.7 months, SD = 2.8, mean age at T2 = 66.7 months, SD = 2.8	Mother reported time spent gaming in the past 7 days	ToM was assessed using six tasks: knowledge access, unexpected contents, real-apparent emotion, Smarties, the Sally Ann first order false belief and second order false belief tasks	At T1, gaming of boys negatively predicted ToM scores at T2. No link between ToM scores and gaming time was observed in girls
Hopp et al. (2018)	Online survey	*N* = 312, 60.77% male, mean age = 32.042, SD = 9.927	VGs played were listed and rated in terms of violence and the frequency played	Eight stories were used. Participants had to rate the behavior and the intent as hostile, instrumental, or benign	No significant relationships were found between exposure to FPS military-themed VGs and hostile attribution bias
Zhen et al. (2011)	Adolescent students in urban China	795 students divided into three groups. Grade 5: *n* = 232, mean age = 11.63, SD = 0.57. Grade 8: *n* = 247, mean age = 14.82, SD = 0.59. Grade 11: *n* = 316, mean age = 16.80, SD = 0.59	VGs played were listed and then rated regarding their violent content and the frequency played	Presented with ambiguous stories, participants had to imagine and write down ten possibilities of what happened next. The number of aggressive possibilities was recorded	Hostile expectations may mediate the relationship between VVG exposure and physical aggression. However, mediation effects were not significant for most of the subgroups (according to grade or sex)
Moller and Krahe (2009)	Secondary school students	3 waves of a longitudinal assessment: T1 (*n* = 295, mean age = 13.34, SD = .83) and T2 (30 months later, *n* = 143)	VVG exposure was assessed with questionnaires regarding VGs played and frequency	Four scenarios were presented, in which participants had to decide if the harm occurred was on purpose	Aggressive norms mediated the link between hostile attribution bias and VVG exposure. Moreover, VVG exposure at T1 seemed to impact aggression (measured by self-report questionnaires) by increasing aggressive norms (measured with a self-report questionnaire) and hostile attribution bias. No significant direct link between VVG exposure and hostile attribution was found
Eastin and Griffiths (2009)	Midwestern University students	*N* = 162, mean age = 21.50, SD = .80, range = 18–41	VG experience was measured by assessing how long participants had played VGs in terms of which frequency and duration	Participants completed three ambiguous stories and had to decide what would happen next and what the character was feeling or thinking (20 answers). Answers were then categorized as aggressive/hostile or nonaggressive/nonhostile. Hostile expectation scores corresponded to the number of hostile answers divided by the total number of answers	VG experience was positively correlated with hostile attribution bias: greater VG experience was associated with higher hostility bias
Gentile et al. (2009) (first of the three studies)	Singaporean secondary school children	7th grade: *n* = 446. 8th grade: *n* = 281. Mean age = 13.0, SD = 0.79	Frequency of gaming, the content of games played and the number of hours per week played were rated	Six ambiguous stories were used, and participants had to explain the event	A negative correlation was found between exposure to prosocial VGs and hostile attribution bias, while VVG exposure was positively correlated with hostile attribution bias
Krahé and Moller (2004)	8th grade students	*N* = 231, mean age = 13.6, SD = 0.63	VVG exposure was measured by self-reports of liked and played games, frequency, and duration of use. The rating of violence was determined by two groups of experts	Four scenarios were proposed in which participants had to rate the hostile intent	No significant correlation between frequency of VVG use and hostile attribution bias was found
Funk et al. (2003)	Children	8- to 12-year-olds: *n* = 35, mean age = 10.14 years, SD = 0.97. 5- to 7-year-olds: *n* = 31, mean age = 5.61, SD = 0.62	Self-reports revealed gaming frequency and content. This study also studied the short-term impact of gaming	Ten drawings depicting stories in which empathy or aggression were induced were read to children. Children had to say what follows (assessing ToM) and how they would react. A higher score for empathy vignettes indicated higher empathy, and a higher score for aggressive vignettes indicated higher aggression	Regression results revealed a negative association between VVG exposure and empathy scores
**Social decision-making (group comparisons)**					
Su et al. (2018)	College students from Fuzhou University, players of “League of Legends”	Problematic Internet game use (PIGU): *n* = 23. Occasional Internet game use (OIGU): *n* = 23	The PIGU group was composed of participants with an IAT score ≥ 50, at least 14 h of Internet gaming per week, with gaming as the principal activity among Internet activities. The OIGU group was composed of participants with an IAT score ≤ 50 and no more than 7 h of gaming per week for at least 1 year	Both economic games were played with a friend, with a recently met experimenter who played with the participant just before, and a stranger. Prisoner’s dilemma (PD): single exposure, participants could give 0 to 10 talers. Talers given were doubled if the other participant cooperated and lost if the other participant did not cooperate. The chicken game (CG): single exposure, the riskiest choice was to defect	No significant difference was found on the PD task, but there was a reduced tendency to cooperate on the CG task in the PIGU group compared to the OIGU group. Both groups were more cooperative with their friends, but only the PIGU group displayed similar levels of cooperative behavior between friends and the game mate just met in the CG task
Jin and Li (2017) (only study three is presented)	Undergraduate students from Sichuan University, China	*N* = 132, 102 men, mean age = 18.93, SD = 2.01, range = 18–21. Experienced players: 67 participants. Inexperienced players: *n* = 67	Groups were composed based on the total gaming exposure. Those in the top 25% comprised the experienced group, and those in the bottom 25% comprised the inexperienced group. Three experimental conditions were used in which two participants performed a task together: 2 players from the same group (experienced or nonexperienced) or one player from each group (experienced and nonexperienced)	Social dilemma task: 10 trials, 1 yen for each trial. Participants could give coins to the partner; money was doubled if the partner cooperated or lost if they did not	Inexperienced gamers tended to be more cooperative than experienced gamers, even when controlling for experimental conditions
**Aggressive behavior**					
	*Group comparisons*				
Pichon et al. (2021)	Same as previously explained in this table	Same as previously explained in this table	Same as previously explained in this table	Study 1: CRT with zero as a possible level of noise blast in Session 1	Study 1: In the CRT, the AVGP group displayed higher level of aggression than the NVGP group
Engelhardt et al. (2011)	Undergraduate students	Age range: 18–22 years	A subgroup of 2000 undergraduates who completed a survey on VG habits (number of hours per week and violent content). One subgroup was randomly selected from the bottom 25th percentile (low-exposure group); the other was selected randomly from the 75th percentile (high-exposure group)	The CRT was used. Participants were told that they would play a rapid game against another participant and that the loser would receive a noise blast with the duration and intensity decided by the winner. In reality, they played against a computer, and intensity and duration chosen were used to a measure aggressive behavior. Participants played a violent or a nonviolent VG and viewed violent or nonviolent photos during EEG recording before the CRT	Participants who played an aggressive VG were more aggressive than the other group (mean = − 0.59), regardless of previous experience. The level of aggression was mediated by desensitization to violence (according to EEG) in low-exposure participants
Arriaga et al. (2011)	College students	*N* = 58, mean age = 22.6, SD = 2.5, range = 18–27	Participants were separated based on violent game habits (VGH, *n* = 30, played VVGs in the past 3 months) and not having a habit of playing violent games (non-VGH; did not play a violent game in the past 3 months, *n* = 28)	The CRT was used. Participants were then interviewed to determine their motivations regarding their answers. Participants were also asked to play a violent or nonviolent VG before	For non-VGH participants, no difference in aggressive behavior was found between before and after playing VGs. For the VGH group, participants who played the VVG displayed more aggressive behavior than participants that did not play the VVG before
	*Correlational studies*				
MacGowan and Schmidt (2021)	Children	Same as previously explained in this table	Same as previously explained in this table	Prosocial behavior was only assessed at T2. It consisted of engineered interactions with the experimenter (one in which the experimenter drops a tape, another in which the experimenter drops paperclips). Latency to help the experimenter was the measure of prosocial behavior	Regarding prosocial behavior, there was no significant association with time spent gaming, in girls or boys
Devilly et al. (2021)	University students and nonstudent gamers	151 participants (141 university students, 10 nonstudent gamers recruited via announcements), 59 males (mean age = 54.44, SD = 8.95) and 92 females (mean age = 22.89, SD = 7.72)	A questionnaire about media usage was used to assess weekly media usage, including type of media (TV, movies, VGs)	The modified Taylor competition reaction time task (TCRTT) was used, with a “no sound” option indicating a nonviolent answer	Media exposure and specifically VVG exposure did not predict the level of aggression on the TCRTT
Ferguson and Rueda (2010)	Young adults from a Hispanic-serving public university	*N* = 103, mean age = 23.6, SD = 2.82	VG habits were assessed with a self-report in terms of the frequency and violent content of VGs	The CRT was used. Before this task, participants played a violent game (high violent or morally acceptable violent games), a nonviolent game or no game at all	No link was found between previous exposure to VVGs and aggression in the laboratory
Ferguson et al. (2008) (first study)	Undergraduate students	*N* = 101, mean age = 20.9, SD = 3.7, range = 18–40	VG habits were assessed with a self-report in terms of the frequency and violent content of VGs	The CRT was used. Before the CRT, participants played the (assigned or chosen) VG, which could be violent or nonviolent	No significant effect of past VG exposure on aggressive behavior was found, and there was no interaction with VG play before the CRT
Bartholow et al. (2006)	Undergraduate students	*N* = 39, mean age = 19.5, only men (only 34 participants were included in the results)	VVG habits were assessed with a self-report in terms of the frequency and violent content of VVGs	The CRT was used. Pictures (violent or not) were also presented while EEG data were recorded	VVG exposure was highly correlated with aggression scores. In the VVG group, the levels of aggression were negatively correlated with the amplitude of the P300 (reflecting information processing, in this study: reaction to violent pictures). A separate regression analysis showed that increased VVG exposure was associated with greater aggression in the CRT Additionally, the greater the difference in P300 amplitudes between violent and nonviolent pictures (with higher reactions to violent pictures), the greater the aggression was. Finally, when separating the frequency and content of the game, only VVG exposure (not the frequency) was correlated with higher aggression scores
Bartholow et al. (2005). Two studies. Only one assessed social cognition with cognitive measures	Undergraduate students	Subset of participants of the other study described in the article (*n* = 92, from 18 to 22 years old)	This study assessed the short-term effect of VG exposure and measured VVG exposure	The CRT was used. A violent or nonviolent VG was played before the CRT	Participants with higher VVG exposure displayed more aggressive behavior than participants with low VVG exposure, especially for those who played a VVG just before the CRT
Tamborini et al. (2004)	College students	*N* = 182, 99 males, mean age = 20.57, SD = 1.52, range = 18–30	VVG and VG exposure in general were assessed with a self-report of the amount of time spent playing VG	Aggressive behavior was assessed by a research assistant. Participants were told that an applicant applied for a position at the laboratory and that they had to rate the courteousness, competency, and worthiness of deserving financial support. Low scores indicated a high level of aggressive behavior	No variable predicted aggressive behavior
**Moral competence**					
	*Group comparison*				
Sofia and Klimenko (2019)	Undergraduate students	*N* = 236, 20% of males	Two subsamples were created from the global sample never/rarely play and play twice or more. The experimental group saw an excerpt of a VG moral dilemma at the end of the semester	The moral competency test (MCT) asked participants to rate the resolution of two moral dilemmas on a scale regarding their agreement with the resolution. A higher score showed high consistency in moral reasoning	A positive correlation between frequency of play and MCT scores was shown; greater frequency of play was reflected in a higher MCT score, but only for those in the experimental condition who had to think about a specific moral dilemma
	*Correlational study*				
Krcmar and Cingel (2016)	University students	*N* = 65, median age = 20	Frequency of VG play in last week and last month was rated	Participants were told to play a VG and had to explain the reasons for each of their choices in the VG. Each choice was coded as strategic choice/reasoning (aimed to improve the character/progress in the game) and/or moral reasoning (irrelevant to progress but reflected morality). For each moral choice, a rating on the foundation behind it was made (harm/care, fairness/reciprocity, in-group/loyalty, authority/respect, purity/respect)	Participants who reported playing more frequently each week were more likely to use moral reasoning during VGs. Moreover, VG use was significantly linked with the foundation of fairness/reciprocity

*CRT* Competitive Reaction Time Task, *EEG* Electroencephalogram, *ERPs* Event-related Brain Potentials, *fMRI* Functional Magnetic Resonance Imaging, *FPS* First-person Shooter, *IAT* Internet Addiction Test (Young, 2016), *IGAS* Internet Game Addiction Scale (Cui, 2006), *GD* Gaming Disorder, *SC* Social Cognition, *MMN* Mismatch Negativity, *SD* Standard Deviation, *ToM* Theory of Mind, *VG* video games, *VVG* Violent Video Games

#### Facial/Social Emotion Processing

Thirteen articles explored the link between exposure to VGs and social emotion processing. Cerebral processing was assessed in some of these studies. For example, the articles compared the performance of two groups of players of violent VGs (frequent or infrequent players) on the stop signal task (SST) and recorded event-related potentials (ERPs). The unconscious processing of emotional facial stimuli was different for happy faces, with smaller amplitudes observed in frequent players (Stockdale et al., 2017). Regarding the detection of emotional stimuli, research has shown that after 10 h of training on VGs (action or nonaction), differences in reaction times are observed between the nonaction VG group and control group, and the type of VG played had influences the impact on cerebral processing (Bailey & West, 2013). Finally, seven studies assessed the ability to process and discriminate facial emotions in gamers without PG. Studies have shown that years of experience with VGs could improve emotion identification and that the number of hours played may negatively impact joy identification (Miguel et al., 2017). Exposure to VGs may also be linked with worse ability to identify negative emotions (Miedzobrodzka et al., 2021a). Moreover, VG players seemed better at recognizing fear but worse at recognizing disgust than controls (Diaz et al., 2015). Additionally, a study found that professional e-sport players and controls displayed similar abilities in identifying identical emotions; both were lower than the capacity of professional baseball players. In the same study, the authors showed that elite e-sport players displayed faster reaction times than the rest of the group (Kang et al., 2020). Finally, no difference appeared between VG players and non-VG players in the identification of morphing emotions (Pichon et al., 2021). Using the reading the mind in the eyes task (RMET), no link between violent VGs and RMET scores was found in participants after 8 weeks of VG practice (Kühn et al., 2019). With the same task, a positive link between the ability to infer correct mental states and better VG (MOBA) performance was observed (Bonny et al., 2020).

In comparisons of participants with PG and controls on tasks assessing unconscious processing of faces (Peng et al., 2017) and cartoons and faces (He et al., 2019), studies have shown differences in ERPs. For example, one study showed a reduction in the N170 component, which reflects the detection of a face (Schweinberger & Neumann, 2016), in the PG group when processing neutral faces compared to happy faces. There was no reduction in the control group. Another study showed a larger peak in response to cartoons faces than in response to human faces in only the right hemisphere in the PG group, while this difference was present in both hemispheres in the control group. These results suggest specific alterations in the processing of facial stimuli in PG (see Table 3 for details). Moreover, when comparing PG patients to controls or to patients with methamphetamine use disorder on a task of social perception, both patient groups displayed lower scores than the control group (Jiang et al., 2020). Finally, a higher number of symptoms of PG were linked with worse scores on the RMET (Aydın et al., 2020).

#### Empathy

Five studies assessed empathy in a population of gamers. No differences were found regarding empathy between frequent and nonfrequent gamers, using an empathy-for-pain task with emotional stimuli (photography or drawings) and functional magnetic resonance imaging (fMRI) (Gao et al., 2017; Szycik et al., 2017). No significant changes were observed, after an 8-week period of exposure to VGs (Kühn et al., 2018). However, comparisons of individuals with low exposure to violent VGs with individuals with a high level of exposure revealed a desensitization to painful stimuli (i.e., similar EEG results for painful and nonpainful stimuli) in only the high-exposure group (Miedzobrodzka et al., 2021b). Finally, no link between violent VG exposure and a scale measuring empathy for a rape victim was found (Ferguson & Colwell, 2020).

No study was identified that assessed empathy in PG.

#### Theory of Mind/Bias of Attribution

Nine studies explored ToM in relation to VG exposure. Among them, eight studies explored ToM by means of stories. Studies with young children showed no link between the rate of aggressive intent in ambiguous stories and exposure to VGs (Ferguson & Wang, 2019). Nevertheless, when using vignettes in which participants had to tell what was going on next but also how they would have reacted in stories that were designed to induce empathy, a significant link between higher exposure to violent VGs and lower scores was reported (Funk et al., 2003). Studies with adolescents showed a negative correlation between exposure to prosocial VGs and hostile attribution bias as well as a positive correlation between hostile attribution bias and exposure to violent VGs (Gentile et al., 2009). Moreover, one study did not find a relationship between exposure to VGs and attribution bias (Krahé & Möller, 2004). Finally, attribution bias was identified to mediate the relationship between exposure to VGs and behavior (Möller & Krahé, 2009; Zhen et al., 2011). Studies with adults showed a positive correlation between exposure to VGs and levels of aggressive thoughts, feelings, and behaviors attributed to characters in ambiguous stories (Eastin & Griffiths, 2009). However, no link between attribution bias and frequent exposure to FPSs was reported (Hopp et al., 2018). Finally, in a study of children that used six different tasks to assess ToM, gaming time negatively predicted boys’ scores on the ToM task after a year (MacGowan & Schmidt, 2021).

#### Social Decision-Making

One study assessed social decision-making in undergraduates relative to their experience with VGs. This study showed that experienced gamers tended to be less cooperative than nonexperienced gamers (Jin & Li, 2017).

One study assessed social decision-making in PG using the chicken game paradigm. The chicken game is an economic game in which two players have to simultaneously make a decision. In this paradigm, not cooperating is the riskiest option but has the highest gain. PG patients tended to take riskier options and cooperate less than controls (Su et al., 2018). Moreover, PG patients displayed comparable levels of cooperative behavior when playing either with a friend or a game mate who they just met in the chicken game task. In contrast, controls were more cooperative with only their friend.

#### Aggressive Behavior

Nine studies explored the link between the frequency of playing VGs and the competitive reaction task (CRT) which is designed to explore aggressive behavior. In this task, participants are instructed to react as fast as possible to a noise stimulus. The loser (i.e., the slowest individual) is punished by a blast of noise, with the duration and intensity of noise are decided by the winner. In most experiments, tasks are programmed to provide half of the victories to the participant, and the intensity and duration of the blast of noise are measures of aggressive behavior (Chester & Lasko, 2019; Taylor, 1967). These studies have shown mixed results. A higher level of exposure to violent VGs has been linked to a more aggressive response pattern (Bartholow et al., 2005, 2006) and specific EEG responses in reaction to violent pictures (Bartholow et al., 2006). Interestingly, in this last study, the authors separated the effect of content (violent or not) from the frequency of play, and the link with aggressive behavior was significant only in relation to the content. Furthermore, action VG players displayed a higher level of aggression than nonplayers (Pichon et al., 2021). Moreover, a tendency to display greater aggression in the CRT after playing a violent VG was found, regardless of previous gaming experience (Engelhardt et al., 2011). Nevertheless, among a population of regular gamers, this link was found only in the group that played a violent VG and not in the group that played a neutral game (Arriaga et al., 2011). Finally, no links were found in four other studies (Devilly et al., 2021; Ferguson & Rueda, 2010; Ferguson et al., 2008; MacGowan & Schmidt, 2021).

Another type of measure was used in the last study: participants were asked to rate the profile of an applicant for a paid position in the laboratory in terms of competence, courtesy, merit of financial support, and merit of employment. Lower ratings were considered a measure of aggressive behavior. No link was found between exposure to VGs and the level of aggressive behavior (Tamborini et al., 2004).

No study was identified that assessed aggressive behavior in PG.

#### Moral Competence

Two studies assessed moral competence in a population of students and showed a positive link between the exposure to VGs and moral reasoning during VG play (Krcmar & Cingel, 2016). Moreover, exposure to VGs was also correlated with moral competence (Sofia & Klimenko, 2019).

No study was found on moral competence and PG.

## Discussion

This article aimed to review all studies assessing SC with neuropsychological tasks NPG and PG. Thirty-nine articles that examined several aspects of SC were identified. Regarding *facial emotion processing*, the results of conscious facial emotion processing were mixed, but the results of unconscious and cerebral processing suggested that a change in processing emotions can in participants with PG. However, the variety of methods and populations assessed did not allow us to conclude that better or worse abilities were linked to VG use or PG. The results involving *empathy* mostly showed no differences linked to the use of VGs. *Attribution bias* studies had mixed results and assessed multiple populations (children, adolescents, and adults). *Social decision-making* studies reported a change in the processing of social distance (assessed when playing with friends or strangers just met in the game) in the PG population. Studies also showed less cooperative behavior in the adult population linked to NPG and PG. Regarding *aggressive behavior*, the results were mixed, and further research is needed. Nevertheless, half of the studies did not find a link between exposure to VG and CRT scores. Studies with PG are lacking. Finally, two studies showed that higher *moral competence* when presented with a moral dilemma was linked to higher VG exposure.

### Gaming and Face Processing

Numerous studies were reviewed in this article, which shows the interest in and the importance of understanding the link between facial emotion processing and gaming. Nevertheless, the neuropsychological data did not clearly indicate deficits or improvements in identifying facial emotions but rather suggested cerebral changes in facial emotion processing. PG patients exhibit deficits in EEG studies and more precisely in the P300 component, which reflects attention allocation (Kuss, 2018). Moreover, it has been shown that excessive Internet users also display altered early processing of faces in terms of ERPs (He et al., 2011). Thus, studies have suggested changes in the cognitive processing of faces. These changes need to be confirmed. The preservation of emotion identification also needs to be confirmed with other studies in regular NPG and PG.

Three studies used the RMET to assess the ability to identify complex facial emotions based on pictures of eyes (Kittel et al., 2022). One study showed no effect of VG exposure (Kühn et al., 2019). Another study identified a link between negative emotion recognition and PG (Aydın et al., 2020). Finally, one study demonstrated a positive association between improved emotion recognition and MOBA competencies (Bonny et al., 2020). Nevertheless, these results should be interpreted with caution because the psychometric properties of the RMET appear to be fragile (Higgins et al., 2022; Pavlova & Sokolov, 2022). Indeed, one limitation of studying SC is that cognitive tasks are mainly experimental and statistically weak. There is no consensus regarding the assessment of SC in the general population. Nevertheless, this limitation has been overcome in the framework of schizophrenia. Indeed, researchers have studied several components of SC and identified tools to assess them, proposing a gold standard of SC assessment in schizophrenia. The social cognition psychometric evaluation (SCOPE) study concluded that the hinting task (hint understanding), the Penn Emotion Recognition Test (ER40, facial emotion recognition) and the Bell Lysaker Emotion Recognition Task (BLERT, facial/vocal/body emotional identification) were the tools with the strongest psychometric properties and should be used in clinical trials (Pinkham et al., 2018). Replicating such work in other pathologies or for the general population may improve assessments of SC and understanding of the SC in gamers.

### Gaming and Empathy

Regarding empathy, no effects of VGs were found in studies using fMRI and diverse cognitive tasks. The interpersonal reactivity index (IRI (Davis, 1983)) is a self-report questionnaire that comprises fantasy and perspective-taking subscales (two measures of cognitive empathy) and empathic concern and personal distress subscales (two measures of affective empathy). A study that used to IRI to assess problematic Internet users found no stable associations (Melchers et al., 2015). However, when another questionnaire, the empathy quotient (Baron-Cohen & Wheelwright, 2004), was used, a link was found between lower levels of empathy and higher problematic Internet use (Melchers et al., 2015). Furthermore, no differences were found when comparing PG and NPG players (Collins & Freeman, 2013). These data suggest that PG is not associated with a diminution of empathy. Nevertheless, more studies are needed to reach conclusions regarding the profile of empathy among gamers and specifically those with PG. For example, no study has explored the affective and cognitive components of empathy, such as those proposed in the substance use disorders population with the Condensed and Revised Multifaceted Empathy Test (MET-CORE) (Grynberg et al., 2017). This study showed an alteration in the cognitive ability to decode complex emotions but preservation of sharing abilities (affective component, as assessed by self-reports cited herein). Thus, further studies are needed to explore whether PG is associated with the same patterns of alterations as those observed with substance use disorders. If this is the case, it would suggest that alterations in cognitive empathy are linked to addiction per se and not to the ingestion of neurotoxic substances.

Finally, in the general population, it has been shown that asking participants to feel empathy while in the fMRI scanner during an empathy-for-pain task created a bias for painful faces, with an overestimation of the intensity on faces (Naor et al., 2020). This study showed that SC components such as empathy and facial emotion processing are not discrete functions and that they have to be measured together to evaluate their reciprocal impacts.

### Gaming, Theory of Mind, and Aggressive Behavior in the General Aggression Model (GAM)

Studies assessing *ToM* and VGs mainly focused on attribution bias and showed mixed results, while *aggressive behavior* was mainly assessed with the CRT and yileded mixed results. For those two SC components, correlational methods have mainly been used, making interpretation more complex and less powerful. Two studies suggested that immediately after having played violent VGs, there is an increase in aggressive behavior, but only for frequent gamers (Bartholow et al., 2005, 2006). This aspect has not been explored in the PG population. This field of research was developed in relation to the general aggression model (GAM) (DeWall et al., 2011). This model postulates that repeated exposure to violent VGs impacts cognition by increasing aggressiveness and then increasing aggressive behavior (Greitemeyer & Mügge, 2014). In our review, 18 out of 31 studies found significant results when assessing the impact of exposure to violent VGs. Moreover, several meta-analyses explored the impact of violent VGs on aggression showing that violent VGs seem to be linked to aggression (Anderson et al., 2010; Bushman & Huesmann, 2006; Prescott et al., 2018). Other meta-analyses have shown no effect of VGs on aggressive behavior (Elson & Ferguson, 2014; Ferguson and Kilburn, 2010; Sherry, 2001). Another meta-analysis reported nuanced these results and as VGs impacted social outcomes (behavioral, cognitive, affective and arousal measures, all mixed in the analysis) by increasing aggressive cognitions and decreasing prosocial outcomes (violent VGs) and increasing prosocial outcomes and decreasing aggressive cognitions (prosocial VGs) (Greitemeyer & Mügge, 2014). The variety of the methods used in the reviewed studies prevents firm conclusions, highlighting the complexity of the relationships between violent VGs and aggressive behavior. More research is needed, and future studies should consider using a thorough assessment of VG habits together with a wide ranging assessment of aggressive behavior (using both cognitive tasks and self-report measures).

Studies on aggressive behavior are absent from the PG literature, and this topic needs further research.

Additionally, aggression is rarely cited in theoretical models of SC, as it may be considered a biased behavior rather than a cognitive ability. Nevertheless, aggression can also be conceptualized as a non-prosocial behavior that falls under the spectrum of SC.

### Gaming and Social Decision-Making

Studies assessing VG experience with and without addiction showed a tendency for reduced cooperation by experienced players. In PG, the study by Su et al. (2018) showed less overall cooperation during the chicken game, suggesting a preference for risky situations or less cooperative behavior. Specific patterns of responses were also found in nonsocial decision-making tasks in this population, with the same level of preference for risky decision-making (Dong & Potenza, 2016). Moreover, PG subjects tended to prefer short-term rewards (Dong & Potenza, 2014). Therefore, it is not clear whether these subjects choose riskier situations or display less cooperation per se.

Finally, during the chicken game (Su et al., 2018), PG players tended to cooperate with game mates as much as they did with friends and cooperated less often with occasional players. These results suggest that game mates were viewed as friends. PG participants, therefore, seem to present a specific pattern of relationships that may be altered in real life but not online.

Social decision-making has been explored in relation to PG and NPG, and research has shown that players’ decision-making seems to be marked by decreased cooperation. Nevertheless, the pattern of decision-making in individuals with PG is a tendency to choose the riskiest options (Schiebener & Brand, 2017), explaining this lack of cooperation. More research is needed to determine whether the tendency to cooperate is altered or impacted by the tendency to make riskier decisions.

### Gaming and Moral Competency

Regarding moral competence, both studies described in this review suggested an improvement in moral competence associated with VG frequency, highlighting a positive impact of VGs (Krcmar & Cingel, 2016; Sofia & Klimenko, 2019). The authors linked these results to the fact that players used to violent VGs tended to more easily emotionally connect with the character presented in the moral dilemma (Sofia & Klimenko, 2019).

More research is needed to determine whether VG players show higher moral competence than nongamers and to explore this aspect in the PG population.

### Impact of SC as a Whole

Only four studies of 39 used more than one task to assess SC, thus preventing conclusions regarding on an overall SC profile among video gamers. Such an overall profile can only be investigated by using a combination of measures, which may facilitate investigation of links between several components of SC. Thus, there is a need for studies to assess SC in video gamers using a combination of measures to assess a combination of SC components. Such multimodal assessment may also provide useful insight into the management of PG, by enabling therapists to focus treatment on the specific functions that are altered.

Additionally, SC may be linked with social functioning in gamers or with the motive to play. Indeed, the profile of SC may be different depending on what is looking for in VG by gamers. Complementary studies including levels of loveliness and motivation to play may allow us to examine the bigger picture of SC in gamers.

### Problematic Gaming and SC

Interestingly, all studies including PG participants found significant results when assessing SC abilities. Indeed, when presented with facial emotions, PG subjects displayed unique cerebral functioning in two studies (He et al., 2019; Peng et al., 2017). Compared to controls, PG patients displayed a deficit in social perception similar to that of patients with methamphetamine use disorder (Jiang et al., 2020). Additionally, lower scores on the RMET were linked to Internet PG symptoms (Aydın et al., 2020). Finally, PG patients displayed lower cooperation than control participants who played a VG (Su et al., 2018). These deficits were be linked with alterations at the on a cerebral level in adolescents (Schettler et al., 2022) and adults (Yan et al., 2021) with PG. Specifically, hyperconnectivity of networks supporting affective processes (limbic structures and cortex networks) and hypoconnectivity of structures supporting cognitive control (such as frontoparietal areas) have been highlighted (Yan et al., 2021). Finally, studies assessing children and adolescents must be considered carefully because the brain and other cognitive functions are still under development and must be considered in transition (Kilford et al., 2016).

On the other hand, no massive effect of exposure to VGs appeared in the experimental literature. It would be interesting to assess addictive symptoms as a function of VG frequency or exposure to determine if the differences observed in this literature search, specifically those that show alterations in SC, are linked with an addictive profile. Indeed, as explained in Bandura’s model, psychosocial functioning can be explained as individual factors, such as cognitive functioning, reciprocally interacting with the environment and behavior (Bandura, 1986). In the context of VG use (behavior), the presence of alterations in SC (individual factor) may be a vulnerability factor that leads to addictive disorders. Reciprocally, the repetition of behavior and time spent playing VGs may lead to alterations in SC. In the same way, social capital or relationships (the environment) reciprocally interact with VG use and alterations in SC. More longitudinal studies are needed to answer these questions.

### Limitations

#### Limitations of the Included Studies

Most included studies had a small sample size and assessed students from universities, which is a recruitment bias. Moreover, most research assessing the effects of long-term exposure to VGs has not assessed addictive symptoms, yet the presence of addictive symptoms can bias the results. Additionally, all included articles assessed only one component of SC and therefore did not permit the generation of SC profiles of participants, which could allow us to examine the links among SC components. Finally, most studies used correlation methods or group comparisons, but no longitudinal study was included, which does not allow us to make conclusions about the causality of identified links.

#### Limitations of the Review

Several limitations arose from the articles reviewed in this article. In particular, heterogeneity was the main characteristic of the research included. Indeed, variations in participants included age groups, definitions of PG (based on either international classifications — GD/IGD — or not), and gaming intensity; methods used and components assessed did not allow comparison between studies or generalization of results. Given this heterogeneity, this review did not include a meta-analysis, and therefore, we could not draw conclusions regarding specific alterations or preservations of cognitive functioning. Moreover, SC is a wide concept that could benefit from a consensus about which components should be included and how to assess them. Regarding the information collected about PG, the main limitation was the lack of homogeneity in the methods used, which highlights the necessity to conduct new research examining PG and SC.

### Future Directions

This review showed that VGs have been investigated through the prism of SC but with many different methods and populations, leading to mixed results. Nevertheless, studies examining PG and SC remain scarce. Understanding SC abilities in VG players with or without addiction is essential to (i) better understand mechanisms of addiction and (ii) better inform the treatment of patients with PG by developing specific cognitive remediation programs that focus on specific alterations in SC. Questions that remain unexplored are as follows: (i) whether offline relationships are impaired and whether this impairments is explained by deficits in SC or withdrawal from offline relationships because of an overinvestment in online relationships; (ii) whether the difficulties related to offline relationships are present before the development of PG, leading these individuals to play and socialize online (i.e., an adaptive strategy), or conversely, are triggered by the high amount of VG use, which cuts these individuals off from real-life relationships and provokes online investment as compensation; and (iii) whether levels of SC deficits relate to levels of alterations observed in the PG population (specifically in social spheres such as school/work, family or friends). The recent recognition of PG can facilitate the development of research and therapies.

## Data Availability

Data and code sharing is not applicable to this article as no datasets were generated or analyzed during the current study.
